# Metabolic reprogramming: the central mechanism driving inflammatory polarization in rheumatoid arthritis and the regulatory role of traditional Chinese medicine

**DOI:** 10.3389/fimmu.2025.1659541

**Published:** 2025-09-18

**Authors:** Jianting Wen, Jian Liu, Lei Wan, Fanfan Wang, Yang Li

**Affiliations:** ^1^ Department of Rheumatology and Immunology, First Affiliated Hospital of Anhui University of Chinese Medicine, Hefei, Anhui, China; ^2^ Institute of Rheumatology, Anhui Academy of Chinese Medicine, Hefei, Anhui, China; ^3^ Anhui Province Key Laboratory of Modern Chinese Medicine Department of Internal Medicine Application Foundation Research and Development, Hefei, Anhui, China

**Keywords:** rheumatoid arthritis, metabolic reprogramming, inflammatory polarization, traditional Chinese medicine, glucose metabolism reprogramming

## Abstract

Rheumatoid arthritis (RA) is characterized by high morbidity, disability, and mortality rates and is intricately linked to metabolic reprogramming that governs immune cell dysfunction and inflammatory polarization, thereby driving RA pathogenesis. This review systematically explored the impact of metabolic dysregulation (especially in glucose, lipid, mitochondrial, and glutamine metabolism) on shaping the inflammatory microenvironment of RA. Key metabolic axes included aerobic glycolysis (the Warburg effect), *de novo* fatty acid (FA) synthesis, mitochondrial bioenergetic dysfunction, and glutaminolysis. Furthermore, the review highlighted the therapeutic potential of traditional Chinese medicine (TCM) in modulating these metabolic pathways to attenuate pro-inflammatory responses and ameliorate RA progression. Through regulation of metabolic enzymes, TCM demonstrated multi-faceted efficacy in restoring metabolic homeostasis and inhibiting pathological inflammation. This review underscored that metabolic reprogramming was pivotal for identifying novel therapeutic targets; our results may provide a scientific foundation for integrating TCM into RA management strategies. These findings advocated for further exploration of metabolism-centered interventions to develop precision therapies for RA.

## Introduction

Rheumatoid arthritis (RA) is a chronic autoimmune disorder characterized by persistent synovial inflammation and progressive joint destruction, greatly impairing patients’ quality of life (1–3). It has been shown that inflammatory response is a pivotal driver in RA pathogenesis (4, 5). As has been evidenced previously, the activation of diverse immune cells in the RA synovium triggers the release of pro-inflammatory mediators, initiating and sustaining a self-amplifying inflammatory cascade, thereby significantly promoting disease development and progression (6–8).

**Figure 1:**
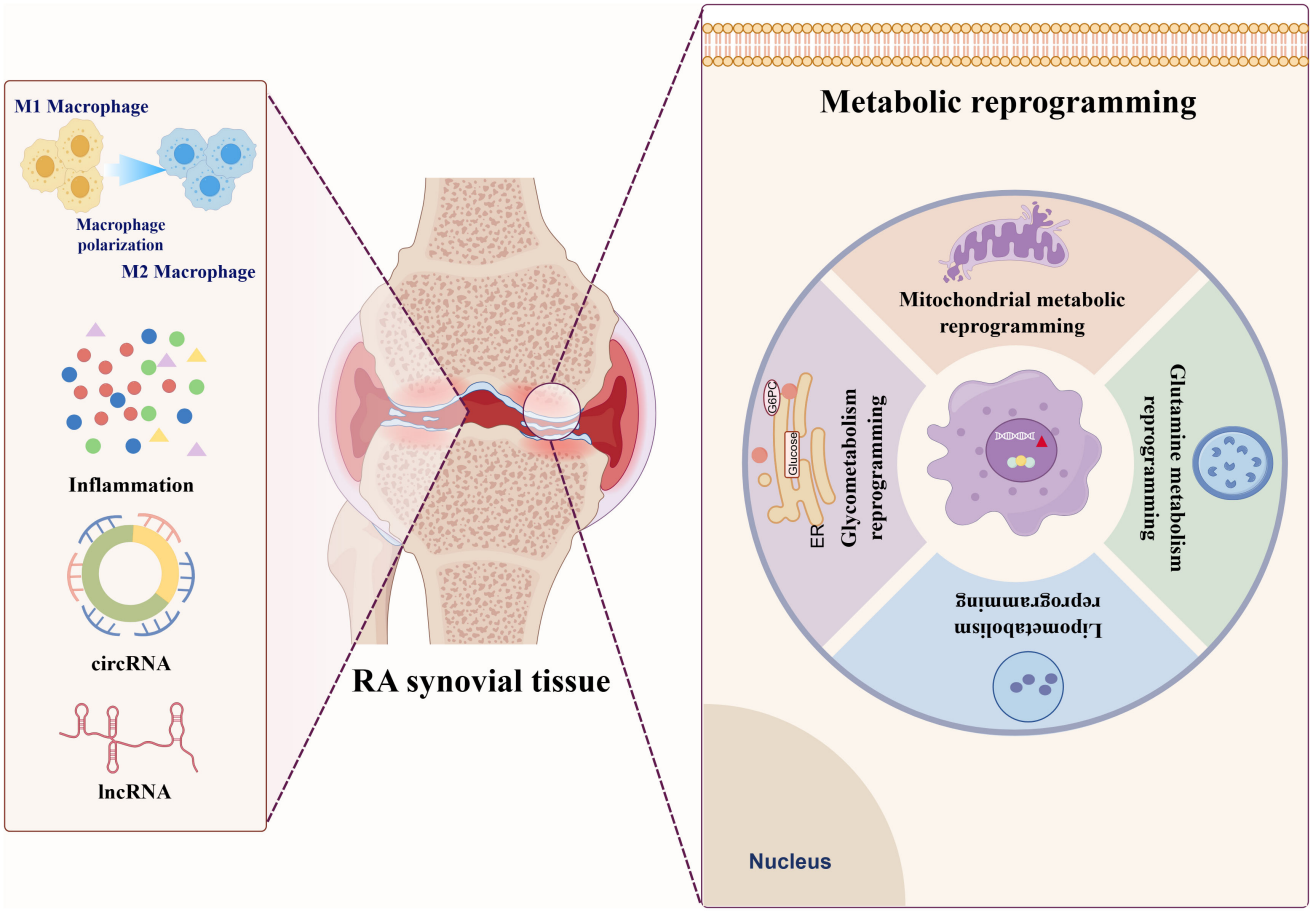
Distinct patterns of metabolic reprogramming in RA Glucose metabolism reprogramming in RA.

**Figure 2:**
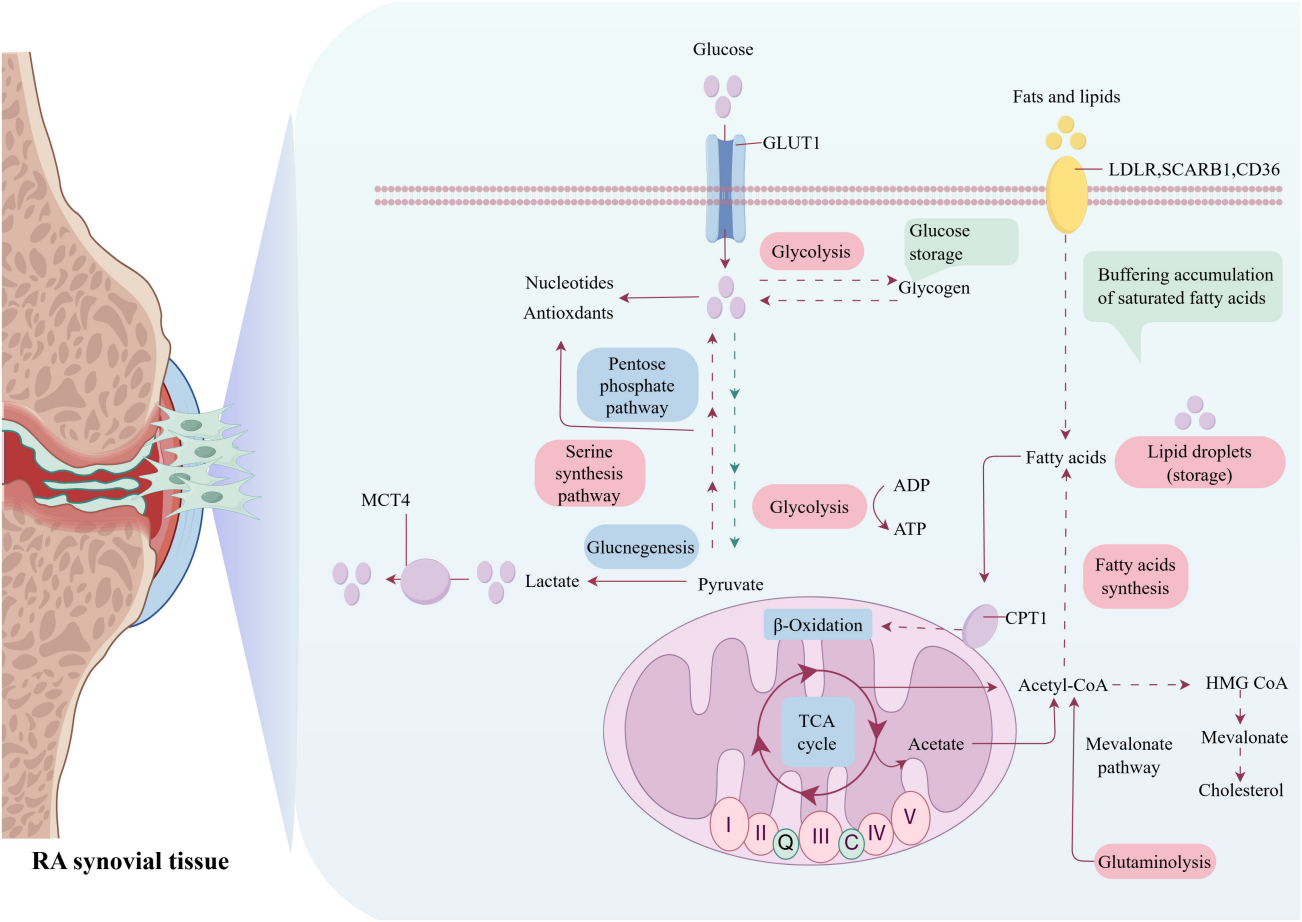
Metabolic reprogramming in RA: interplay of glycolytic, lipogenic, mitochondriogenic, and glutaminolytic pathways.

Metabolic reprogramming is a critical biological process in which cells alter their metabolic patterns to meet the energy requirements for growth and proliferation, which serves as a fundamental driving factor in the pathogenesis and progression of RA (9, 10). This process is characterized by profound alterations in core metabolic pathways, including glycolysis, FA synthesis, mitochondrial dysfunction, and glutaminolysis. These adaptations not only meet the requirements of rapidly proliferating RA immune cells for biosynthesis and bioenergy, but also actively promote inflammatory response (11). Crucially, metabolic crosstalk among RA immune cells notably accelerates disease progression. During RA pathogenesis, immune cells dynamically modulate their metabolic profiles to ensure essential nutrients, enabling sustained proliferation within the hypoxic and nutrient-deprived synovial microenvironment. Additionally, these metabolic alterations induce significant phenotypic and functional changes in RA immune cells, leading to a self-reinforcing state of reprogramming (12). This reprogrammed metabolic state ultimately empowers these cells to permanently experience inflammation and actively promote RA progression (**Figures 1**, **2**).

Although recent advances in RA metabolism research have yielded significant insights into these pathogenic mechanisms, there are considerable challenges in translating this knowledge into effective metabolic pathway-targeted therapies. Specifically, these challenges include pathway redundancy, complexity of metabolic crosstalk within the inflammatory milieu, and potential off-target effects. In contrast, traditional Chinese medicine (TCM) offers a distinct therapeutic paradigm, grounded in its foundational principles of a “holistic perspective” and “syndrome differentiation and treatment” (13). This therapeutic perspective inherently aligns with the multifaceted nature of RA pathogenesis. Namely, TCM interventions may exert therapeutic effects on RA through multi-pathway, multi-target, and multi-angular mechanisms. Specifically, accumulating evidence has indicated that TCM formulations and bioactive compounds can modulate key metabolic axes dysregulated in RA, including lipid homeostasis, glucose utilization, and amino acid metabolism (notably glutaminolysis) (14). By rectifying these metabolic perturbations, TCM interventions could ameliorate synovitis and systemic inflammation, thereby mitigating disease progression. This metabolic modulation represents a crucial mechanism underpinning the therapeutic efficacy of TCM in RA management.

This review explored the metabolic reprogramming in RA, with a focus on glucose, lipid, mitochondrial, and glutamine metabolism. The present research not only discussed how these reprogrammed pathways promoted inflammatory polarization and RA progression, but also investigated how TCM can modulate this reprogramming to suppress inflammation and exert therapeutic effects. The review aimed to provide novel therapeutic targets and strategies for RA treatment from a metabolic perspective.

## Glucose metabolism reprogramming: key enzymes and pathways

Glucose metabolism reprogramming denotes a fundamental adaptive shift in cellular energy metabolism, characterized by a preferential augmentation of aerobic glycolysis (the Warburg effect), pentose phosphate pathway (PPP) flux, and hexosamine biosynthetic pathway (HBP) activity even under normoxic conditions (15). This reprogramming is coordinated by dysregulation of key metabolic enzymes, including hypoxia-inducible factor 1α (HIF-1α)-induced HK2, phosphofructokinase-2/fructose-2,6-bisphosphatase 3 (PFKFB3), pyruvatebkinase M2 (PKM2), and lactate dehydrogenase (LDHA), as well as increased glucose transporter 1 (GLUT1) (16, 17). Critically, this metabolic shift is not merely a passive consequence but an active driver of inflammation through multiple interconnected mechanisms.

Growing evidence has supported that metabolic reprogramming underpins chronic inflammation in autoimmune diseases. For example, it has been demonstrated that in RA patients, toll-like receptor 2 (TLR2) expression on CD4^+^ T cells is increased and positively correlated with C-reactive protein (CRP) and rheumatoid factor (RF) levels. Mechanistically, TLR2 activation induces changes in glucose metabolism-related enzymes within CD4^+^ T cells, leading to a shift in glucose flux towards the PPP (18). These findings indicate that inflammation and glucose metabolic reprogramming interact bi-directionally in RA, and the inflammatory milieu can drive metabolic shifts towards glycolysis in resident cells within the joints, thereby exacerbating RA-associated inflammation (19, 20). Accumulated TCA cycle intermediates, such as succinate and fumarate arising from anaplerotic fluxes (including glutaminolysis and mitochondrial dysfunction), stabilize HIF-1α and activate the NLRP3 inflammasome. Lactate acidifies the microenvironment, thus promoting the secretion of pro-inflammatory cytokines [e.g., interleukin (IL)-1β, IL-6, and tumor necrosis factor (TNF)-α] and inhibiting anti-inflammatory functions. Additionally, PPP-derived NADPH maintains reactive oxygen species (ROS) generation and redox-sensitive signaling (e.g., NF-κB activation). HBP-generated UDP-GlcNAc promotes O-GlcNAcylation of NF-κB subunits and histones, enhancing the expression of inflammatory genes. Lastly, inflammatory cytokines (e.g., TNF-α and IL-1β) further activate HIF-1α and NF-κB, reinforcing the expression of glycolytic enzymes and establishing a self-amplifying inflammatory-metabolic circuit.

## Glucose metabolism reprogramming drives inflammatory polarization in RA

Abnormal glucose metabolism is a common and significant pathological feature in RA, which is closely linked to systemic inflammation (21). Accumulating studies have provided evidence that glycolytic enzymes are significant markers of abnormal glucose metabolism. For example, Pi et al. have discovered novel differentially expressed enzymes implicated in the glycolysis pathway in a collagen-induced arthritis (CIA) rat model, such as hexokinase and fructose-bisphosphate aldolase (22). Moreover, as indicated by the data from Ahn et al., plasma PKM2 levels in RA patients are significantly positively correlated with erythrocyte sedimentation rate (ESR), disease activity score in 28 joints (DAS28), and CRP (23). These findings suggest that plasma PKM2 may serve as a clinically useful biomarker candidate for assessing disease activity. The abnormal expression of these glycolytic enzymes is not only associated with inflammation in RA, but may also serve as a new target for RA diagnosis and treatment (**Table 1**).

**Table 1:** Key metabolic pathways, targets, and metabolite alterations involved in RA pathogenesis.

Metabolic reprogramming	Targets	Models/cells	Metabolites	Mechanisms
Glucose metabolism reprogramming	/	CIA rats	Hexokinase and fructose-bisphosphate aldolase	Increase apoptosis (22)
	/	Plasma	↑Lactate	Evaluate disease activity and RA diagnosis (23)
	α-Taxilin	RA-FLS	PKM, DHA	Mediate inflammation (24)
	eEF2K	RA-FLS	F2,6BP	Suppress glycolysis and aggressive behaviors of RA-FLS (25)
	TNF/TAK1	RA-FLS	↑Glucose uptake/ ↑Lactate	Induce glycolytic shift in RA-FLS (26)
	SAE1/UBA2	CIA mice and human TNF-α transgenic mice	↑Lactate	Suppress glycolysis, aggressive phenotype, and inflammation (27)
	AMPK/NF-кB	AA rats	↑Lactate	Alleviates bone destruction, inhibit the proliferation and migration of FLS, and reduce secretory function of FLS (28)
	lncRNA TUG1/miR-34a-5p	RA-FLS	↑Lactate	Promote glucose metabolism and apoptosis resistance (29)
	c28MS	RA-FLS	↑Lactate	Upregulate glycolysis and glutaminolysis (30)
	RBM15/NLRP3	CIA mice	CXCL9, CXCL10	Reduce macrophage glycolysis and inhibit NLRP3 inflammasome activation (31)
	LXRs/AMPK/mTOR	AIA	↑Lactate	Protect against bone erosion by inhibiting M1 macrophage polarization (32)
	AMPK/SIRT1	AIA	↑Lactate	Impair the inflammatory polarization of monocytes (33)
	AMPK/NF-кB	AIA	ECAR, OCR	Inhibit macrophage polarization (34)
	IL-34	FLS	↑Glucose uptake/ ↑Lactate	Broaden the inflammatory and metabolic phenotypes (35)
	IFN-γ/IL-17	M1MφS	↑Glucose uptake/ ↑Lactate	Disrupte glycolysis (36)
	IRAK4/miR-LET7B/TLR7	RA-MφS, RA-FLS	↑Lactate	Disrupte glycolysis (37)
	SIRT3	CD4+ T cells	↑Fructose-2,6-bisphosphate	Decrease glycolysis, reduce ATP production, and induce apoptosis in CD4+ T cells (38)
	TNF-α/ITK-Akt-mTOR	CD4+ T cells	ECAR, OCR	Drive metabolic reprogramming (39)
Lipid metabolism reprogramming	LKB1-AMPK	FLS	Fatty acid β	Promote the proinflammatory (40)
	AZGP1	HFD-CIA mice		Accelerate inflammation and fat loss (41)
Mitochondrial metabolism reprogramming	MCU	CIA mice	Mitochondrion	Inhibit RA-FLS migration (42)
	IL-17	RA-FLS/Th-17 cell	Mitophagy	Increase autophagy of RA-FLSs by causing mitochondrial dysfunction (43)
	MST1/SIRT3/mTOR	RA-FLS	Mitophagy	Reduce mitochondrial autophagy and promote mitochondrial division (44)
	MST1/AMPK/SIRT1	RA-FLS	Mitophagy	Promote mitochondrial dysfunction and apoptosis (45)
	TL1A/TNFR2	RA-FLS	Mitochondrion	Mediate mitochondrial dysfunction amplify the inflammatory response (46)
Glutamine metabolism reprogramming	/	RA-FLS/SKG mice	↑Glutamate	Inhibite RA-FLS proliferation (47)
	lncRNA NEAT1/miRNA-338-3p	RA-FLS	GLS	Promote glutamine metabolism and resistance to apoptosis (48)

Moreover, glucose metabolic disturbances in RA patients may also be associated with immune imbalances. Immune cells [such as fibroblast-like synoviocytes (FLSs), T cells, and macrophages] play crucial roles in RA, with their high energy demands resulting in the accumulation of metabolic byproducts and inflammatory mediators. These molecular events affect RA-related effector cells (including circulating immune cells and joint-resident cells), thereby exacerbating systemic inflammation and promoting the ongoing progression of joint lesions. Sarkar et al. have reported that α-Taxilin drives RA pathogenesis by interacting with glycolytic enzymes to induce metabolic dysregulation, ROS production, and TLR-mediated inflammation in RA-FLS (24). Additionally, as indicated by Chen et al., eEF2K is a critical regulator linking glucose metabolism reprogramming to aggressive FLS phenotypes in RA, and is proposed as a novel therapeutic strategy (25). GLUT1 and HIF1A play crucial roles in glucose metabolism reprogramming, especially in inflammation-related disease processes. Specifically, GLUT1, as the primary glucose transporter, promotes glucose uptake and metabolism in various cell types. HIF-1α induces the expression of glucose transporters and glycolytic enzymes that promote glucose uptake and glycolysis. For instance, Koedderitzsch et al. have revealed that TNF-α directly reprograms glycolytic metabolism in RA-FLS through the GLUT1/HIF1A axis (26). Furthermore, multiple studies have confirmed that aggravated inflammation and accelerated glycolysis are tightly implicated in RA. For example, Wang et al. have suggested that elevated expression of SUMO-activating enzyme SAE1/UBA2 in RA-FLSs and synovial tissues promotes joint inflammation through SUMOylation-dependent metabolic reprogramming (27). Another study has demonstrated that glycolysis is involved in pathological inflammation in RA joints; glycolysis inhibition may ameliorate RA by suppressing synoviocyte activation via the AMPK/NF-κB pathway (28). Therefore, blocking glycolysis may be beneficial for RA, and exploring critical regulatory nodes linking glycolytic metabolism and inflammation may contribute to RA treatment. Notably, lncRNAs, acting as a competitive endogenous RNA (ceRNA) to regulate miRNAs, play significant roles in RA glucose metabolism. For instance, Zhang et al. have elucidated a key ceRNA network involving lncRNA TUG1 and miR-34a-5p, which regulates glycolytic metabolism and apoptosis resistance in RA-FLSs through targeting LDHA (29). Research has increasingly suggested that targeting more than one metabolic pathway may be a novel therapeutic approach for inflammatory diseases. According to data from Ahmed et al., the dual inhibition of glycolysis and glutaminolysis by compounds (such as c28MS) effectively targets pathogenic metabolic reprogramming in RA-FLS, suppressing their aggressive phenotype and ameliorating experimental arthritis (30).

Accumulating studies have provided evidence that glucose metabolism reprogramming is important in macrophage phenotype transition. For instance, Yu et al. have reported that the m6A enzyme RBM15 mitigates RA damage by reducing macrophage glycolysis and inhibiting NLRP3 inflammasome activation (31). Zheng’s team has demonstrated that liver-X-receptor (LXR) inverse agonist SR9243 can effectively slow adjuvant-induced arthritis (AIA) progression by inhibiting M1 macrophage polarization and activation, which is primarily achieved through regulating glycolytic metabolism, with specific mechanisms involving the modulation of the AMPK/mTOR/HIF-1α signaling pathway (32). Additionally, AMPK and SIRT1 are key molecules that regulate cellular metabolism and inflammatory responses. It has been shown that the deficiency of AMPK and SIRT1 could lead to abnormal activation of the glycolytic pathway in the AIA, promoting monocyte inflammatory polarization (33). This polarization not only exacerbates arthritis severity but also affects the overall immune response and metabolic balance. Furthermore, in support of this, Cai et al. have observed that the anti-arthritis effects of 2-DG are mediated through AMPK-dependent regulation of macrophage polarization (34). IL-34 is closely linked to the worsening of RA. Van Raemdonck et al. have confirmed that IL-34 reprograms the glycolysis and osteoclast activity of RA macrophages by binding to the M-CSFR and SDC-1, and facilitates their cross-regulation with T effector cells (35). Umar et al. have demonstrated that RA macrophages (MΦs) and FLSs exhibit distinct metabolic reprogramming during inflammation (36). Although both cell types utilize the PPP, RA M1-type MΦs primarily rely on glycolysis, whereas RA-FLSs engage both glycolysis and mitochondrial oxidative phosphorylation (OXPHOS). Critically, this study concluded that 2-DG was therapeutically superior to inhibiting OXPHOS in RA, largely due to its potent suppression of the pro-inflammatory MΦ phenotype that is central to RA pathogenesis. Similarly, a previous study has noted that IRAK4 inhibition (IRAK4i) mitigates RA joint inflammation by reprogramming metabolic dysfunction in synovial macrophages and FLSs (37). Taken together, the aforementioned studies have suggested that targeting the glycolytic pathway and concomitant inflammatory phenotype in synovial MΦs and FLS may be a crucial therapeutic approach for rebalancing metabolic dysregulation in RA.

The dysregulation of T cell glucose metabolism, especially the pathological reprogramming towards a hyperglycolytic phenotype, has emerged as a critical focus in understanding RA pathogenesis. Sirtuin 3 (SIRT3) plays a critical role in regulating T cell metabolism during RA pathogenesis. Wang et al. have revealed that SIRT3 deficiency impairs glycolysis, reduces ATP production, and induces apoptosis in CD4^+^ T cells, thereby exacerbating AIA in mice (38). Another study has indicated that TNF-α signals through ITK-Akt-mTOR to drive CD4^+^ T cell glucose metabolism reprogramming in RA, which suggests that target components of this signaling axis may thus represent a potential therapeutic strategy for modulating T cell glucose metabolism in RA (39).

## Lipid metabolism reprogramming in RA

### Lipid metabolism reprogramming: key enzymes and pathways

Lipid metabolism reprogramming is defined as pathological changes in cellular lipid synthesis, uptake, storage, and catabolism pathways, representing a fundamental adaptive mechanism underlying the pathogenesis of various diseases (including cancer, metabolic disorders, and chronic inflammatory conditions) (40). Its dysregulation arises from complex mechanisms involving aberrant activation of key transcriptional regulators (e.g., SREBPs, LXRs, and PPARs), oncogenic signaling cascades (e.g., PI3K/AKT/mTOR and HIF-1α), and microenvironmental stressors. Critically, lipid metabolism reprogramming elevates specific bioactive lipid metabolites, which function as potent signaling molecules and pathological drivers (41). The metabolic products of lipid metabolism primarily include: phospholipids (e.g., phosphatidic acid and lysophospholipids) that modulate membrane dynamics and mitogenic signaling; sphingolipids (e.g., sphingosine-1-phosphate and ceramides) that govern cell survival, death, and migration; and eicosanoids (e.g., prostaglandins and leukotrienes) that are derived from arachidonic acid. These metabolites exert profound pro-inflammatory effects by activating receptors (e.g., S1PRs, LPARs, and prostaglandin receptors) on immune and stromal cells, thereby amplifying the production of various cytokines (e.g., TNF-α, IL-6, and IL-1β) and chemokines (42). Reciprocally, chronic inflammatory signaling directly interferes with lipid metabolic enzyme expression and activity, forming a sustained feed-forward loop that promotes disease progression and tissue damage. Consequently, it’s crucial to understand the intricate interplay between lipid metabolism reprogramming and inflammation to identify novel therapeutic targets.

Immune cells undergo metabolic reprogramming during differentiation and activation to meet their energy demands. As an important component of cell membranes and an energy source, lipid metabolism directly affects the function of immune cells, leading to immune cell dysfunction and subsequently promoting the onset and progression of chronic inflammation in RA (43). Lipid metabolites can affect RA progression by regulating the activity and function of immune cells (e.g., T cells and FLSs). It has been shown that the levels of 12-22C saturated FA, palmitoleic acid, oleic acid, and linoleic acid in RA-FLS are higher compared with those in OA-FLS (44). Moreover, when stimulated with free FA, RA osteogenesis secretes more pro-inflammatory cytokine IL-6, chemokine IL-8, growth-related oncogene-α, and MCP-1. This indicates that the association between lipid metabolism and immune cells in RA has recently received considerable attention.

### Lipid metabolism reprogramming drives inflammatory polarization in RA

RA patients often suffer from multiple metabolic disorders, which are closely linked to lipid metabolism dysregulation. By integrating multi-omics data, significant changes have been found in the lipid metabolism pathways in RA patients, particularly in cholesterol and FA metabolism (45). These changes not only affect RA pathological progression, but also offer new insights into the early diagnosis and treatment of RA-related metabolic diseases. Additionally, Jiang et al. have identified 22 potential differential FAs-associated biomarkers in serum and 13 differential FAs-associated biomarkers in synovial fluid in CIA model through microdialysis combined with lipidomics, including hexadecanoic acid, octadecanoic acid, arachidonic acid, DHA, DPA, myristic acid, and succinic acid (46). Moreover, there is a complex relationship between the lipid profiles of RA patients and disease activity. Analysis of the plasma lipid profiles of 278 RA patients has revealed that FAs (such as stearic acid and palmitic acid) are negatively correlated with disease activity, suggesting that these lipids may contribute to RA pathogenesis by affecting bone metabolism (47).

The role of increased n-6 polyunsaturated fatty acids (PUFAs) in the synovium of RA is complex, with both pro-inflammatory and anti-inflammatory lipid regulatory effects. Comparative lipidomic analysis of synovial tissue has revealed that relative to OA patients, RA patients display a lower proportion of SFAs and a higher proportion of longer-chain SFAs, MUFAs, alkylacyl chains, and C20 omega-6 PUFAs (48). Therefore, in-depth exploration of the dual role of n-6 PUFAs in inflammation could offer new insights into RA diagnosis and treatment. Fatty acid β-oxidation (FAO) regulates cell function. Recent breakthroughs in RA have demonstrated that leptin-enhanced FAO activates AMPK by upregulating LKB1, thus promoting the pro-inflammatory characteristics of RA-FLS. Additionally, IL-17 and Th17 cells are tightly implicated in the pathogenesis of RA, and their involvement is especially pronounced under high-fat diet (HFD) conditions (49). Na et al. have suggested that Th17 and IL-17 accelerate inflammation and fat loss by inducing AZGP1 in CIA mice with HFD (50). Similarly, Liu et al. have also confirmed that a HFD could stimulate dysbiosis of butyrate metabolism, alter gut microbiota, and exacerbate inflammatory responses in CIA rat models (51). Another study has also demonstrated that the imbalance of long-chain FAs mediated by gut microbiota increases the proportion of Th17, thereby exacerbating the autoimmune disease psoriasis (52). Hence, HFD could affect the composition of intestinal microbiota and metabolites through multiple mechanisms, thus exacerbating inflammation and subsequently promoting RA progression.

## Mitochondrial metabolism reprogramming in RA

### Mitochondrial metabolism reprogramming: key enzymes and pathways

Mitochondrial metabolism reprogramming represents a fundamental adaptive mechanism in which cells dynamically alter their bioenergetic and biosynthetic pathways to meet the evolving physiological or pathological demands (53). This reprogramming process is characterized by measurable shifts in key metabolic indicators, including oxygen consumption rate (OCR), extracellular acidification rate (ECAR), ATP/ADP ratio, NAD^+^/NADH equilibrium, and accumulation of TCA cycle intermediates (e.g., citrate, succinate, and fumarate) (54, 55). Crucially, mitochondrial metabolism reprogramming is orchestrated by core regulatory pathways (such as the HIF axis, AMPK/mTOR signaling, and sirtuin-mediated deacetylation cascades), which collectively modulate pyruvate dehydrogenase (PDH) activity, ETC efficiency, and anaplerotic flux (56). Importantly, emerging evidence has underscored a bidirectional relationship between mitochondrial metabolic reprogramming and inflammation (57). Pro-inflammatory stimuli (e.g., TNF-α and IL-1β) can induce mitochondrial fission, impair OXPHOS, and promote aerobic glycolysis. However, conversely, metabolic aberrations (such as succinate-driven RET at Complex I or mtDNA release due to mPTP opening) could enhance NLRP3 inflammasome activation, NF-κB signaling, and pro-inflammatory cytokine secretion. Therefore, mitochondrial metabolic rewiring not only sustains cellular homeostasis but also functions as a pivotal node in immunometabolic cross-talk, contributing to the pathogenesis of chronic inflammatory disorders (such as RA).

Mitochondrial metabolism reprogramming plays a crucial role in RA pathogenesis. Regulating mitochondrial function and metabolic pathways may offer new insights and strategies for RA management. Future research should further explore the specific roles of mitochondria in different cell types to develop more effective drugs for RA therapy.

### Mitochondrial metabolism reprogramming drives inflammatory polarization in RA

Mitochondrial metabolism reprogramming is pivotal in RA pathogenesis, driving inflammatory responses and facilitating disease progression. Recent evidence has revealed mitochondrial dysfunction in RA PBMCs, involving reduced ATP, disrupted ΔΨm, elevated ROS, and upregulated ROMO1/OMA1 expression (58). Notably, this analysis did not establish causal links. Hence, it’s necessary to conduct longitudinal studies to validate whether mitochondrial defects occur before the clinical manifestation of RA. Another study has demonstrated that the mitochondrial trafficking facilitator Miro1 mediates the inflammation and invasiveness in RA-FLS by binding to the mitochondrial calcium uniporter (MCU), making it a promising therapeutic target for inhibiting RA progression (59). Mitochondrial autophagy is a selective process that clears damaged or dysfunctional mitochondria, maintaining mitochondrial homeostasis and reducing ROS and damage-associated molecular patterns (DAMPs)-caused excessive inflammatory responses. Previous research has indicated that by inducing mitochondrial dysfunction, IL-17 activates autophagy, thereby affecting RA-FLS apoptosis and promoting inflammatory responses (60). Furthermore, a separate investigation into mitophagy in RA has demonstrated that MST1 suppresses the SIRT3/mTOR signaling, leading to attenuated mitophagy and enhanced mitochondrial fission, thereby reducing inflammation, proliferation, and invasiveness of RA-FLSs (61). Interestingly, several pieces of evidence have shown that autophagy is closely associated with oxidative stress. For example, Wang et al. have found that oxidative stress (H_2_O_2_-induced) upregulates MST1 expression in RA-FLSs, triggering mitochondrial dysfunction and apoptosis (62). MST1 activation suppresses the AMPK-SIRT1 axis, resulting in reduced mitochondrial membrane potential. Crucially, MST1 silencing rescues SIRT1 expression, ameliorating mitochondrial defects and cell death. This study identified MST1 as a key mediator of oxidative damage and mitochondrial dysfunction in RA-FLSs. Additionally, TL1A can influence the behavior of RA-FLSs through the TNFR2 receptor, leading to mitochondrial dysfunction and increased ROS production, thereby exacerbating the inflammatory response (63).

Taken together, mitochondrial metabolism reprogramming not only plays a key role in RA pathophysiology, but may be a promising potential target for future treatment of RA. Delving into the mechanism of mitochondrial metabolic reprogramming contributes to providing new ideas and methods for the treatment strategy of RA.

## Glutamine metabolism reprogramming in RA

### Glutamine metabolism reprogramming: key enzymes and pathways

Glutamine metabolism reprogramming represents a hallmark adaptation in which cells reconfigure glutamine utilization to maintain biosynthetic precursors, redox homeostasis, and energy production under pathophysiological stress (64). This rewiring is quantifiable through key metabolic indicators: elevated glutamine uptake (via transporters SLC1A5/ASCT2), increased flux through glutaminase (GLS)-dependent deamidation, accumulation of α-ketoglutarate (α-KG) and glutamate, and altered NADPH/NADP^+^ ratios and glutathione (GSH) synthesis rates (65). The key regulatory axes in glutamine metabolism reprogramming include the MYC-driven transcriptional activation of glutamine catabolic enzymes, mTORC1-mediated potentiation of anabolic pathways, and suppression of α-KG dehydrogenase by hypoxia-inducible factor (HIF)-1α, which collectively diverts glutamine carbon toward nucleotide synthesis (purine/pyrimidine), FA production (via reductive carboxylation), and TCA cycle anaplerosis (66).

Notably, glutamine metabolism reprogramming engages in a dynamic crosstalk with inflammation. For example, it has been shown that pro-inflammatory signaling (e.g., TNF-α/NF-κB and LPS/TLR4) upregulates GLS and glutamine transporters, promoting macrophage M1 polarization and inflammasome activation through ROS-driven IL-1β maturation (67). Reciprocally, glutamine-derived metabolites modulate immune responses; α-KG regulates Treg/Th17 differentiation through epigenetic mechanisms. GSH depletion exacerbates the activation of NLRP3 inflammasome by mitochondrial ROS. This metabolic-immunological interplay positions glutamine flux as a pivotal determinant of chronic inflammation and autoimmune disease (such as RA) (68).

### Glutamine metabolism reprogramming drives inflammatory polarization in RA

Glutamine metabolism reprogramming closely participates in RA pathogenesis. As has been evidenced previously, glutaminase 1 (GLS1), the first and rate-limiting enzyme of glutaminolysis, exhibits upregulated expression in RA-FLSs (69). Inhibition of RA-FLS cell growth occurs under glutamine deprivation conditions, but not under glucose deprivation conditions. Furthermore, GLS1 inhibition suppresses RA-FLS proliferation, highlighting its potential as a novel therapeutic target for RA associated with glutamine metabolism. Furthermore, the lncRNAs-mediated ceRNA mechanism also regulates glutamine metabolism in RA-FLSs. Zhang et al. have demonstrated that lncRNA NEAT1 enhances glutamine metabolism in RA-FLSs by sponging miR-338-3p, thereby relieving its inhibitory effect on the target gene GLS; this highlights that lncRNA NEAT1-driven metabolic reprogramming is a promising therapeutic target for RA (70).

### TCM-based interventions on RA through metabolic reprogramming

**Figure 3:**
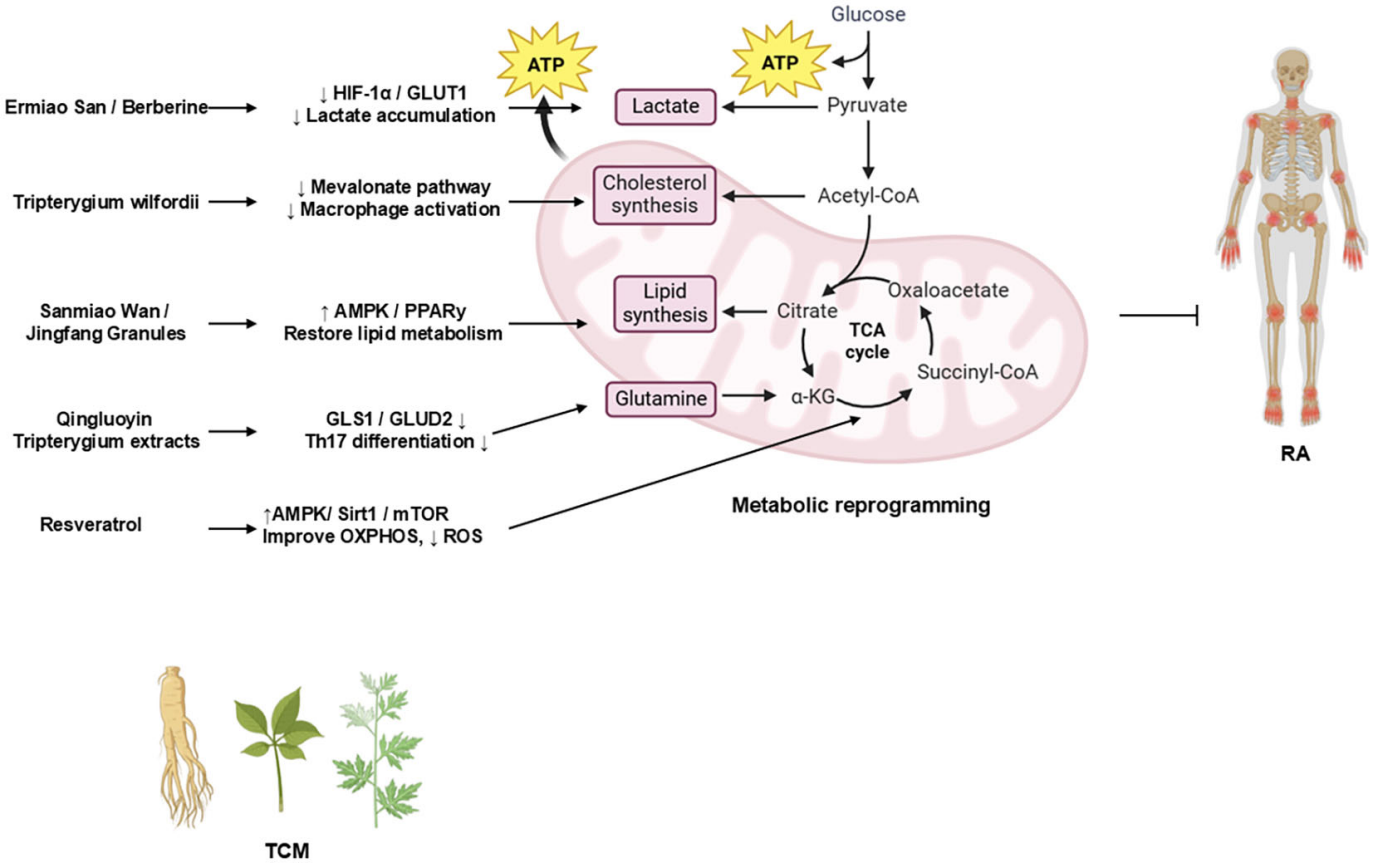
TCM regulation of metabolic pathways in RA.

Intriguingly, TCM has garnered increasing attention for its potential therapeutic effects in RA (71–92). Accumulating preclinical studies have suggested that modulating aberrant metabolic reprogramming represents a key mechanism underpinning the efficacy of various TCM compounds and formulations (**Table 2**). Investigating this TCM-mediated metabolic regulation provides a promising avenue for developing novel RA therapeutics. **Figure 3** summarizes how TCM interventions regulate glucose, lipid, mitochondrial, and glutamine metabolism to suppress RA inflammation.

**Table 2:** Representative TCM formulations and bioactive compounds for modulating metabolic reprogramming in RA.

Metabolic reprogramming	TCM	Objects	Targets	Mechanisms
Glucose metabolism reprogramming	Ermiao San	CIA mouse	PI3K/AKT/mTOR/HIF-1α	Attenuate RA through PI3K/AKT/mTOR signaling activate HIF-1α induced glycolysis (71, 72)
	Qingre Huoxue Decoction	CIA mice	FBP1/AMPK	Reduce the disease activity, attenuate the inflammatory response, and delay bone destruction in RA (73, 74)
	Additive Sishen Decoction	Macrophages	PI3K/AKT/GLUT1/LDHA	Anti-inflammatory responses (75)
	Ginsenoside compound K	AA rats	NF-κB/HIF-1α	Ameliorate AA by inhibiting the glycolysis of FLS through the NF-κB/HIF-1α pathway (77)
	Cepharanthine	Macrophage	LRS-MYD88/IRAK4-IRF5	Attenuate joint inflammation by suppressing monocyte chemotaxis and proinflammatory differentiation (78)
	Berberine	CIA mice	mTORC1/HIF-1α	Ameliorate CIA mice by restoring macrophage polarization by AMPK/mTORC1 pathway switching glucose metabolism reprogramming (79)
	Roburic acid	Macrophages	ERK/HIF-1α/GLUT1	Effectively ameliorates RA symptoms by Metabolic reprogramming of proinflammatory macrophages (80)
	Sarsasapogenin	RA-FLS	PKM2	Attenuate proliferation and invasion in RA-FLS through downregulating PKM2 inhibited pathological glycolysis (81)
	Triptolide	CIA mice	PKM2	Represse the PKM2-mediated glycolysis and attenuate joint inflammation (82)
	Resveratrol	HUVEC	Rho/ROCK/VEGF	Inhibit glycolysis-fueled angiogenesis under RA conditions (83)
	α-Mangostin	HUVEC	VEGF	Inhibite aerobic glycolysis in AIA rats, consequently ease inflammation-related hypoxia, and hamper pathological neovascularization (84)
Lipid metabolism reprogramming	Sanmiao Wan	AA rats	TNF/IL-6/MMP3	Treat RA by inhibiting pro-inflammatory cytokines and modulating both glycerophospholipid metabolism and sphingolipid metabolism (86)
	Sanmiao Wan	AIA rats	Hippuric acid, pyridoxine, and pantothenic acid	Treat RA mainly by reducing inflammation and regulating abnormal lipid metabolic pathways (87)
	Jingfang granules		AMPK	Modulate gut microbiota to activate AMPK, inhibite ferroptosis caused by lipid oxidative stress in synovium tissue and prevente AR injury (88)
	Qing-Luo-Yin	AIA mouse	SIRT1/PPARγ	Up-regulate PPARγ in AIA mice, leading to inflammation remission (90)
	*Illicium verum*	RA-FLS	Lipid and amino acid metabolism	Regulate lipid and amino acid metabolism, then modify inflammation in RA (92)
	α-Mangostin	AIA rats, adipocytes, macrophages	PPAR-γ	Disrupte adipocytes-mediated metabolism-immune feedback (93)
Mitochondrial metabolism reprogramming	Cantleyoside	RA-FLS	AMPK/SIRT 1/NF-κB	Enhance mitochondrial dysfunction by cantleyoside confine inflammatory response and promote apoptosis (94)
	Resveratrol	AA rats	/	Alleviate inflammatory injury and enhance the apoptosis of RA-FLS by mitochondrial dysfunction and ER stress (95)
	Resveratrol	RA-FLS	/	Reduce autophagy derived mitochondrial dysfunction and promote RA-FLA apoptosis (96)
Glutamine metabolism reprogramming	*Tripterygium hypoglaucum*	AA rats	GLUD2	Dock with GLUD2 (97)

The schematic diagram illustrates how representative TCM formulations and bioactive compounds modulate metabolic reprogramming in RA (98). Ermiao San (EMS) and berberine inhibit glycolysis via the HIF-1α/GLUT1 axis, thereby reducing lactate accumulation. Sanmiao Wan and Jingfang Granules restore lipid homeostasis by activating the AMPK/PPARγ signaling. *Tripterygium wilfordii* extracts inhibit the Mevalonate pathway to suppress macrophage activation. Resveratrol improves mitochondrial oxidative phosphorylation (OXPHOS) and reduces ROS production through the AMPK/SIRT1/mTOR signaling. Qingluoyin and *Tripterygium* extracts downregulate GLS1/GLUD2, thereby inhibiting Th17 differentiation. Collectively, these TCM-mediated effects restore metabolic balance and mitigate inflammation in RA.

## TCM-based interventions on RA through glucose metabolism reprogramming

### TCM formulations/prescriptions

By regulating sugar metabolism, TCM can not only meet the energy needs of immune cells but also inhibit excessive inflammatory responses, thereby improving the condition of RA patients. The classical herbal formula EMS has exhibited good efficacy and safety in RA treatment, characterized by minimal taste complexity and potent therapeutic efficacy, which has been proven through extensive clinical practice and prior investigations (71). According to recent breakthroughs in EMS, the beneficial effects of EMS in CIA rats can be attributed to the inhibition of glycolysis-related molecules (HK2 and GLUT1) in FLS through the PI3K/AKT/mTOR/HIF-1α pathway (72). Qingre Huoxue Decoction (QRHXD) is a well-established traditional Chinese medicine formula extensively employed in clinical practice and has significant therapeutic effects on RA (73). Furthermore, in a comprehensive proteomics investigation elucidating the therapeutic mechanisms of QRHXD in RA, Zhang et al. have identified a distinct metabolic signature comprising 54 significantly differential metabolites (DMs) (74). As revealed by KEGG analysis results, these metabolic alterations are primarily concentrated in glycolysis/gluconeogenesis and the mTOR signaling pathway. Subsequent validation studies using CIA mice have demonstrated that QRHXD reduces the disease activity of RA, attenuates the inflammatory response, and delays bone destruction by inhibiting FBP1 and activating the AMPK signaling pathway. Additive Sishen decoction (ASSD) is a widely used TCM for RA treatment, which may exert its efficacy from the perspective of glucose metabolism. In support of this, Ren et al. have observed that kaempferol, luteolin, and quercetin are key active ingredients in ASSD responsible for its anti-RA effects (75). Their mechanistic studies have revealed that these compounds significantly suppress LPS/IFN-γ-induced glycolysis in macrophages, which is a critical metabolic shift driving pro-inflammatory activation.

### TCM bioactive constituents

By regulating glucose metabolism pathways, TCM bioactive constituents may effectively inhibit RA progression and alleviate RA symptoms, showing significant potential in RA treatment (76). Ginsenoside compound K (CK), as an active metabolite of ginsenosides, exhibits significant anti-inflammatory and immunomodulatory effects. Wang et al. have reported that CK reduces the production of inflammatory factors by inhibiting the NF-κB and HIF-1α signaling pathways, thereby alleviating the symptoms of AA rats (77). Cepharanthine (CEP) can effectively improve the synovial inflammation and joint destruction in CIA mice. Specifically, CEP can inhibit LPS and IFN-γ-induced macrophage M1 polarization by downregulating the IL-6-JAK/STAT3 signaling, glycolysis, and oxidative phosphorylation. Berberine (BBR) has demonstrated significant efficacy in improving CIA mice, with its mechanism closely linked to restoring macrophage polarization (78). Data from Cheng have indicated that BBR could ameliorate articular inflammation and restore M1/M2 ratio in CIA mice by activating AMPK and switching the glycolysis reprogramming (79). Jia et al. have demonstrated that targeted metabolic reprogramming of macrophages via an innovative nanomedicine approach is a promising therapeutic strategy for RA (80). The roburic acid they delivered effectively ameliorates RA symptoms. Mechanistically, RBA-NPs reprogram M1 macrophages toward the anti-inflammatory M2 phenotype by inhibiting glycolysis via the ERK/HIF-1α/GLUT1 pathway.

Sarsasapogenin, as the primary active component of Zhi Mu total saponins, has been demonstrated to exhibit anti-inflammatory and immunomodulatory effects. PKM2 is a key glycolytic enzyme involved in aerobic glycolysis, which is considered a key therapeutic and drug discovery target for RA. A previous study has shown that Sarsasapogenin effectively inhibits the activity of PKM2 tetramers and glucose uptake, suppressing glycolysis and thereby inducing apoptosis in RA-FLSs (81). Additionally, triptolide (TP) is a diterpene triepoxide extracted from the Chinese herb *Tripterygium wilfordii* Hook F, which has been used in China for many years to treat inflammatory conditions (including RA). As revealed by a previous study investigating TP for RA treatment, TP could inhibit Th17 cell differentiation via suppression of PKM2-mediated glycolysis, thereby alleviating joint inflammation in CIA mice (82).

Synovial angiogenesis is a critical event during RA pathogenesis, promoting the infiltration of inflammatory cell and exacerbating joint damage. TCM bioactive constituents inhibit RA synovial angiogenesis through glycolysis, thereby exerting effects On RA treatment. Resveratrol is a natural polyphenol with multiple biological activities, possessing several pharmaceutical effects such as anti-inflammatory, antioxidant, and antiviral properties. Specifically, it has been shown that resveratrol reduces the production of RA-related angiogenic cytokines and inhibits glycolysis through the activation of SIRT1, thereby weakening the potential for angiogenesis (83). Similarly, Jiang et al. have noted that α-Mangostin (MAN) can reduce HIF-1α-mediated angiogenesis in adjuvant-induced arthritis rats by inhibiting aerobic glycolysis (84). This process involves the reduction of glucose 6-phosphate, fructose 6-phosphate, 3-phosphoglycerate, and phosphoenolpyruvate, as well as the restoration of LDH’s ability to synthesize pyruvate. Altogether, TCM bioactive constituents exert anti-inflammatory and antiangiogenic effects through various mechanisms, demonstrating potential application value in RA treatment.

## TCM-based interventions on RA through lipid metabolism reprogramming

### TCM compounds

By regulating lipid metabolism, TCM can reduce lipid synthesis, promote lipid breakdown, enhance lipid transport, and suppress the secretion of inflammatory factors, thereby exerting positive therapeutic effects on RA (85). Sanmiao Wan is a classic formula for RA treatment, and clinical and experimental study results have demonstrated its therapeutic effects on RA. Wan et al. first reported that based on integrated lipid metabolomics, serum pharmacochemistry, network pharmacology, and experimental validation, the potential mechanism of Sanmiao Wan in treating RA involves reducing the activity of TNF, IL-6, and MMP-3, as well as modulating glycerophospholipid and sphingolipid metabolism, which provides important clinical insights (86). Moreover, as a classical TCM prescription, Sanmiao Wan has been used for effective treatments for RA in the clinic. Through employing untargeted metabolomics, Cao et al. have demonstrated that Sanmiao Wan effectively treats RA by mitigating inflammation and modulating dysregulated lipid and amino acid metabolic pathways, comprehensively elucidating its lipid metabolic mechanism (87). Jingfang granules are a type of TCM known for its significant anti-inflammatory effects. Wang et al. have indicated that Jingfang granules could enhance the levels of acetic acid, propionic acid, and butyric acid in the intestines and serum of RA rats by increasing the abundance of the intestinal microbiota and regulating the number of intestinal bacteria (88). The increase in these short-chain FAs activates AMPK, which regulates FA metabolism and biosynthesis, thereby inhibiting lipid oxidative stress-induced iron toxicity. Furthermore, the therapeutic effects of Jin Teng Qing Bi granules in the RA model are evident in regulating metabolic disorders. Through non-targeted metabolomics analysis, Tang et al. have identified several potential biomarkers closely related to amino acid and lipid metabolism, further elucidating the metabolic regulatory mechanisms of Jin Teng Qing Bi granules in RA treatment (89). Additionally, Qing-Luo-Yin (QLY), a traditional herbal formula, has been investigated for its efficacy in treating AIA in mice. Research has indicated that white adipose tissue (WAT) releases a significant amount of inflammatory mediators, playing a crucial role in RA pathogenesis. The SIRT1 inhibitor in QLY significantly alleviates the inflammatory symptoms of AIA mice by modulating the inflammatory response in WAT. This study suggested that QLY provides an anti-RA therapeutic approach by independently inhibiting SIRT1 activity in WAT (90).

### TCM bioactive constituents

Berberine, as a TCM bioactive constituent, has attracted extensive attention in recent years in the research of regulating lipid metabolism and promoting cell apoptosis. Specifically, it has been evidenced that berberine impairs the formation of the PPARγ-NF-κB transcriptional complex, downregulates CREB and EGR-1 expressions, and consequently suppresses cellular proliferation and inflammatory responses (91). These findings lay a key foundation for further exploring its therapeutic potential in RA management. The fruit of *Illicium verum* is a medicinal and edible resource that have been proven to possess anti-inflammatory properties. A pioneering study by Qin et al. first used UHPLC-HDMS to analyze the effects of star anise extract on RA-FLSs (92), revealing that star anise extract can inhibit the proliferation and migration of these cells and reduce the levels of inflammatory factors (such as TNF-α and IL-6). This suggests that star anise extract may exert its anti-inflammatory effects by regulating lipid and amino acid metabolism pathways. Additionally, MAN is a natural xanthone derivative that demonstrates potent anti-arthritic effects in AIA rats by restoring adipocyte-mediated metabolic homeostasis and disrupting pro-inflammatory immune-metabolism crosstalk (93). Specifically, MAN alleviates AIA by restoring PPAR-γ–driven adipogenesis and reshaping adipokine secretion, thereby breaking the vicious cycle of inflammation and metabolic dysfunction. This review may provide a mechanistic basis for targeting metabolic-immune feedback loops in RA therapy.

## TCM-based interventions on RA through mitochondrial metabolism reprogramming

Mitochondrial metabolism pathways represent an emerging and feasible target for future anti-rheumatic drugs, especially in the context of the mechanistic role of TCM. Gypenoside is a dammarane glycoside, which is the main component of *gynostemma pentaphyllum* and has strong anti-inflammatory effects. As has been evidenced by Bai et al., CA can limit inflammatory responses by enhancing mitochondrial dysfunction and promote RA-FLS apoptosis by activating the AMPK/SIRT1/NF-κB pathway (94). Moreover, resveratrol may also participate in anti-RA by influencing mitochondrial metabolism and regulating the levels of inflammatory mediators. According to Lu et al., resveratrol could alleviate inflammatory injury in AA rats, triggering RA-FLS apoptosis via the mitochondrial pathway and ER stress, highlighting the dual anti-inflammatory and pro-apoptotic mechanisms of resveratrol in RA by regulating mitochondrial metabolism (95). Another study has also confirmed that resveratrol suppresses mitophagy in RA-FLSs, leading to the accumulation of ROS and subsequent induction of apoptosis (96). These evidences suggest that targeting mitochondrial metabolism is a promising emerging therapeutic approach for TCM in treating RA. Consequently, elucidating the precise molecular mechanisms underlying TCM-mediated mitochondrial regulation provides a crucial scientific basis for developing novel mitochondria-targeted therapeutic strategies against RA.

### TCM-based interventions on RA through glutamine metabolism reprogramming

A recent metabolomic and molecular pharmacological study has revealed that *Tripterygium hypoglaucum* (Hutch.) (THH), as a clinically utilized TCM, alleviates RA by targeting the glutamine-glutamate/γ-aminobutyric acid (GABA) cycle (97). In adjuvant-induced RA rat models, THH extract (THHE) notably downregulates serum glutamine levels, while upregulating the level of glutamate, GABA, and α-ketoglutarate, indicating an enhanced conversion of glutamine to glutamate via GLS. Molecular docking has identified glutamate dehydrogenase 2 (GLUD2) as a key target, with 27 bioactive THH components (wilforine, wilfordine) binding strongly to its active site (PDB ID: 6G2U). Moreover, THHE reduces the release of pro-inflammatory cytokines (IL-1α, IL-1β, and MMP-3), and elevates the expression of anti-inflammatory mediators (IL-4 and IL-10). These findings reveal that the glutamine-glutamate/GABA axis and GLUD2 are pivotal targets for RA immunotherapy with reduced side effects, highlighting the anti-inflammatory effects of TCM in treating RA by regulating glutamine metabolism.

## Conclusions and perspective

This review comprehensively reviews the pivotal role of metabolic reprogramming in driving inflammatory polarization and RA pathogenesis. First, metabolic reprogramming serves as a central driver. Dysregulation of core metabolic pathways (such as glycolysis, lipid metabolism, mitochondrial function, and glutaminolysis) is not merely a bystander but also a fundamental driver of RA pathogenesis. Metabolic reprogramming directly promotes the hyperactivation, survival, and inflammatory polarization of key effector cells (FLS, macrophages, and T cells) in the synovial microenvironment. Second, TCM is a multi-targeted metabolic modulator. TCM emerges as a powerful therapeutic paradigm for RA due to its holistic and multi-targeted approach. TCM compounds (e.g., EMS, Qingre Huoxue Decoction, and Sanmiao Wan) and bioactive constituents (e.g., berberine, resveratrol, cepharanthine, Sarsasapogenin, TP, MAN, and Gypenoside) exert significant anti-inflammatory and joint-protective effects primarily through metabolic reprogramming.

In RA, mitochondrial metabolism is tightly interconnected with glucose, lipid, and amino acid metabolism. Glycolysis provides anaplerotic substrates (such as pyruvate and lactate-derived intermediates) to replenish the TCA cycle and support mitochondrial bioenergetics. Fatty acid β-oxidation delivers NADH and FADH_2_ to the electron transport chain, thereby influencing ROS production and inflammatory signaling. Similarly, glutaminolysis sustains anaplerosis and α-ketoglutarate-dependent epigenetic regulation, shaping T-cell and macrophage polarization. These pathways converge on central regulatory hubs (including AMPK, mTOR, HIF-1α, and sirtuins), which integrate metabolic cues to determine inflammatory outcomes. Therefore, mitochondrial dysfunction not only disrupts intrinsic bioenergetics but also propagates systemic metabolic imbalance, thereby amplifying RA pathogenesis.

Despite significant advances, there are still several challenges and opportunities ahead. First, it’s necessary to decipher metabolic heterogeneity and crosstalk. Single-cell metabolomics and spatial transcriptomics should be employed in future research to unravel the metabolic heterogeneity within different synovial cell populations (FLSs, macrophage subsets, T cell subsets, B cells, and endothelial cells) and metabolic crosstalk between them under dynamic disease states (early established RA, remission, and onset). Second, metabolic drivers and consequences should be investigated in the future. Longitudinal studies in pre-RA or very early RA cohorts are crucial to determine whether observed metabolic changes are the primary drivers initiating autoimmunity/inflammation or secondary consequences of autoimmunity/inflammation events. Identifying “metabolic priming” events is key. Third, it’s crucial to bridge the gap to clinical translation. Rigorous validation of metabolic markers (e.g., plasma PKM2, specific lipid species, and mitochondrial DNA) is essential for early diagnosis, disease activity monitoring, and treatment response prediction. Fourth, TCM formulations should be optimized. Rigorous pharmacokinetic/pharmacodynamic studies and randomized controlled trials (RCTs) should be conducted in the future to standardize TCM formulations, determine the optimal dosage, and definitively establish clinical efficacy and safety profiles. Future research should focus on isolating and characterizing the most potent active metabolites responsible for metabolic reprogramming effects.

Fifth, it’s of great significance to explore novel drug delivery. Future research should explore advanced delivery systems (e.g., nanoparticles like RBA-NPs) to enhance the bioavailability and targeted delivery of metabolic modulators (both synthetic and natural compounds) to inflamed joints. Sixth, exploring synergistic approaches is important. Investigating the rational combinations of metabolic modulators (e.g., glycolysis + glutaminolysis inhibitors) or their integration with conventional DMARDs/biologics to achieve synergistic efficacy and overcome pathway redundancy. Future research should focus on exploring the potential of TCM as an adjunctive therapy. Finally, deepening TCM mechanism studies is crucial. Through multi-omics approaches (metabolomics, proteomics, and metagenomics) combined with network pharmacology and molecular docking/validation, future research could comprehensively map the mechanisms by which specific TCM formulas and compounds rewire metabolic networks in RA. It’s crucial to focus on defining precise molecular targets in key pathways (AMPK, SIRTs, PPARγ, mTOR, and HIF-1α).

Taken together, metabolic reprogramming has been unequivocally established as a core hallmark and actionable therapeutic target for RA. The intricate metabolic-inflammatory relationship provides a fertile ground for developing novel mechanism-based interventions. TCM, with its inherent multi-targeted nature, offers a particularly promising avenue for restoring metabolic homeostasis and quenching pathological inflammation. Future research should focus on understanding metabolic heterogeneity, advancing biomarker discovery, optimizing targeted delivery (including TCM), and conducting robust clinical trials, which is crucial for translating the promise of metabolic therapy into tangible benefits for RA patients, paving the way for more effective and potential treatment strategies.
